# Exploration of mobile genetic elements in the ruminal microbiome of Nellore cattle

**DOI:** 10.1038/s41598-024-63951-7

**Published:** 2024-06-06

**Authors:** Camila A. Faleiros, Alanne T. Nunes, Osiel S. Gonçalves, Pâmela A. Alexandre, Mirele D. Poleti, Elisângela C. Mattos, Flavio Perna-Junior, Paulo H. Mazza Rodrigues, Heidge Fukumasu

**Affiliations:** 1 Department of Veterinary Medicine, School of Animal Science and Food Engineering (FZEA), University of São Paulo, Pirassununga, SP 13635-900 Brazil; 2 Department of Microbiology, Institute of Biotechnology Applied to Agriculture (BIOAGRO), Federal University of Viçosa, Viçosa, MG 36570-000 Brazil; 3 Commonwealth Scientific and Industrial Research Organization (CSIRO), Agriculture and Food, Brisbane, QLD Australia; 4 Department of Animal Nutrition and Production, School of Veterinary Medicine and Animal Science, University of São Paulo (FMVZ-USP), Pirassununga, São Paulo 13635-900 Brazil

**Keywords:** Bacteriophages, Microbial genetics

## Abstract

Metagenomics has made it feasible to elucidate the intricacies of the ruminal microbiome and its role in the differentiation of animal production phenotypes of significance. The search for mobile genetic elements (MGEs) has taken on great importance, as they play a critical role in the transfer of genetic material between organisms. Furthermore, these elements serve a dual purpose by controlling populations through lytic bacteriophages, thereby maintaining ecological equilibrium and driving the evolutionary progress of host microorganisms. In this study, we aimed to identify the association between ruminal bacteria and their MGEs in Nellore cattle using physical chromosomal links through the Hi-C method. Shotgun metagenomic sequencing and the proximity ligation method ProxiMeta were used to analyze DNA, getting 1,713,111,307 bp, which gave rise to 107 metagenome-assembled genomes from rumen samples of four Nellore cows maintained on pasture. Taxonomic analysis revealed that most of the bacterial genomes belonged to the families *Lachnospiraceae*, *Bacteroidaceae*, *Ruminococcaceae*, *Saccharofermentanaceae*, and *Treponemataceae* and mostly encoded pathways for central carbon and other carbohydrate metabolisms. A total of 31 associations between host bacteria and MGE were identified, including 17 links to viruses and 14 links to plasmids. Additionally, we found 12 antibiotic resistance genes. To our knowledge, this is the first study in Brazilian cattle that connect MGEs with their microbial hosts. It identifies MGEs present in the rumen of pasture-raised Nellore cattle, offering insights that could advance biotechnology for food digestion and improve ruminant performance in production systems.

## Introduction

Advancements in metagenomics in rumen environments have enabled us to identify and classify the taxonomy and functional capabilities of bacteria that specialize in digesting plant material, which is the primary food source for ruminants. Given the impact of microorganisms on host cattle performance, microbiota modulation is crucial for developing a sustainable and productive livestock system^1^. For instance, feed efficiency is a trait with implications for economic and environmental sustainability^2^ and is strongly associated with host animal microbiota profiles^3–5^. Ruminal methane production, which results from microbial fermentation in the rumen, is another example of trait that can be explained by genetic variation and the composition of the host animal microbiota^6–8^. Approximately 65% of rumen prokaryotic species have been classified and described in taurine breeds^9^; however, less information is available on Indian cattle, which are the majority in the world, especially in Brazil.

Understanding the role of specific phylotypes in the rumen during fermentation and identifying new enzymes through metagenomic analysis can improve biotechnology for food digestion and ruminant performance in production systems^10^. Important and few characterized components of the rumen are the Mobile Genetic Elements (MGEs), they possess great potential for manipulation and biotechnological applications owing to their ability to confer benefits to their hosts and participate in crucial functional modifications of their hosts, carrying auxiliary metabolic genes such as glycosidic hydrolases (GH), which contribute to the breakdown of complex carbohydrates^11^.

In addition, viruses carry genes that contribute positively their hosts in the metabolism of nutrients and stages of the biogeochemical cycles carried out by the hosts^12,13^. They are classified as mobile DNA elements that move within and between bacterial cells through agents, such as plasmids, bacteriophages, and transposons, resulting in the introduction of accessory genes that contribute to the evolution of host cells^14,15^. Solden et al.^16^ reported that viruses play a crucial role in controlling the ecosystem functions in the rumen, they have metabolic interdependence with their microbial hosts, carry genes that can redirect carbon metabolism, and infect dominant microorganisms that degrade carbohydrates.

Early studies on bacteriophages in the rumen revealed that they can eliminate specific bacteria^17^. Recent research has indicated the potential use of lytic phages to regulate microorganisms in the rumen responsible for an increase in acidosis and methane production^18,19^. This suggests that the components in the rumen are interconnected with viruses that infect ruminal bacteria, the microbiome, and the host animal interacting and affecting each other. This can change the ruminal function and the nutritional capacity of the host animal^20^.

Recent studies have employed the Hi-C approach, which captures intimate interactions between bacterial chromosomes within living cells, to investigate MGEs in microbial communities. This method has been used in conjunction with metagenomic sequencing and has proven effective in assembling genomes and uncovering new relationships between hosts and MGEs^21–24^. The objective of this study was to determine the existence of MGEs in the rumen content of Nellore cattle in Brazil, using the Hi-C method to establish a link between these elements and their microbial hosts.

## Results

### Metagenome of bovine rumen content samples

**Figure 1:**
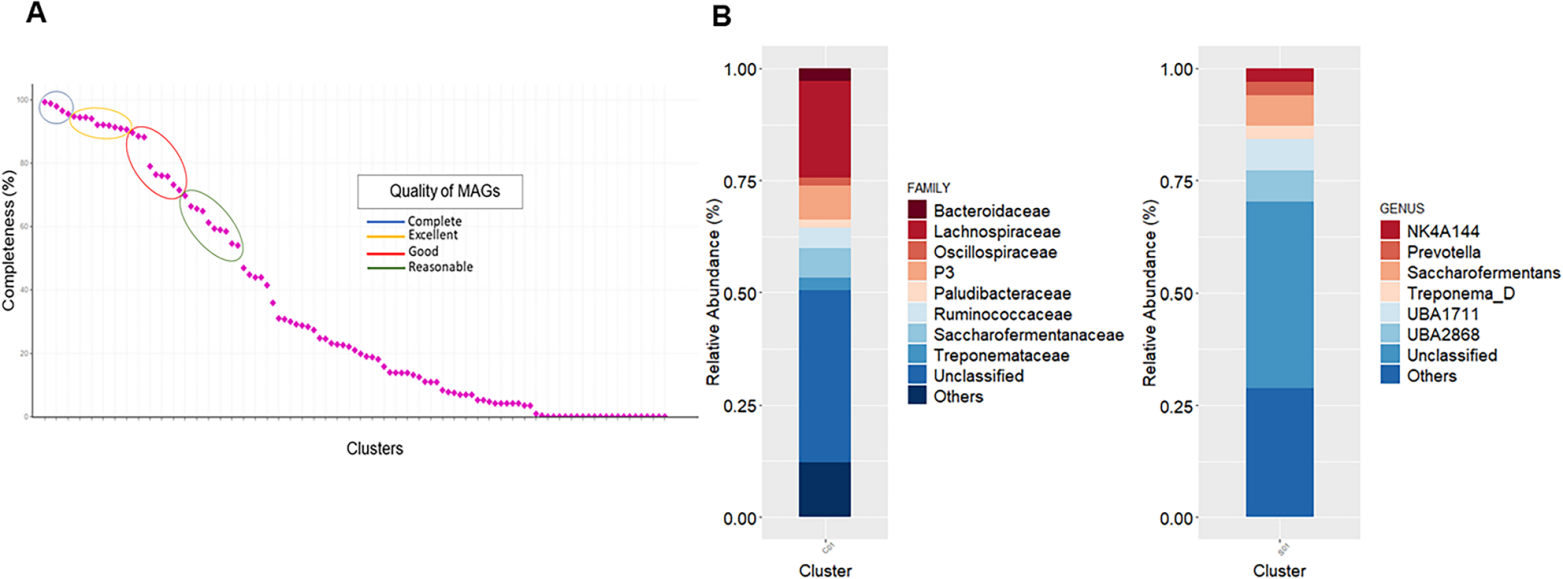
Rumen metagenome of Nellore cattle. (**A**) Completeness of metagenome-assembled genomes (MAGs), (**B**) Taxonomic classification of bins: graph on the left genus and right graph family.

In this study, the metagenomic sequencing was performed with DNA extracted from a pool of rumen content samples of four Nellore cows, applying ProxiMeta, a proximity linkage method based on Hi-C. This method used contigs derived from shotgun sequencing and reads from a library Hi-C, and generated an assembly length of 1,713,111,307 bp, with 834,164 contigs total and 16,035 contigs in clusters allowing the assembly of 107 genomes (Fig. 1A). The most completely assembled genome was bin_4 (99.30%), which corresponded to *Treponema D. sp016288205*. Other genomes classified as complete, with completeness greater than 95%, were *UBA2868 sp003535955* (bin_1), *Treponema D. sp017421145* (bin_9), *Treponema D. sp902783155* (bin_6) and *UBA2868* (bin_12). The clusters classified as excellent (> 90%) were dominated by species from the *Lachnospiraceae* family, namely *UBA3766 sp902803015* (bin_22), *UBA1712* (bin_14), *UBA2868* (bin_3), *UBA2868 sp003535955* (bin_17), *NK4A144 sp902783395* (bin_7), *Acetatifactor sp900066565* (bin_21), *UBA1711 sp900317125* (bin_11), and *UBA1711 sp001543385* (bin_18). Clusters belonging to the families *Saccharofermentanaceae* with *Saccharofermentans* (bin_19), and *Bacteroidaceae* with *Prevotella sp000702825* (bin_5) also had an excellent completeness. The clusters considered good are presented in the Supplementary Table 1, and the 107 clusters subjected to taxonomic classification are represented in Fig. 1B.

### Functional and carbohydrate-active enzymes (CAZYmes) characterization of bins

**Figure 2:**
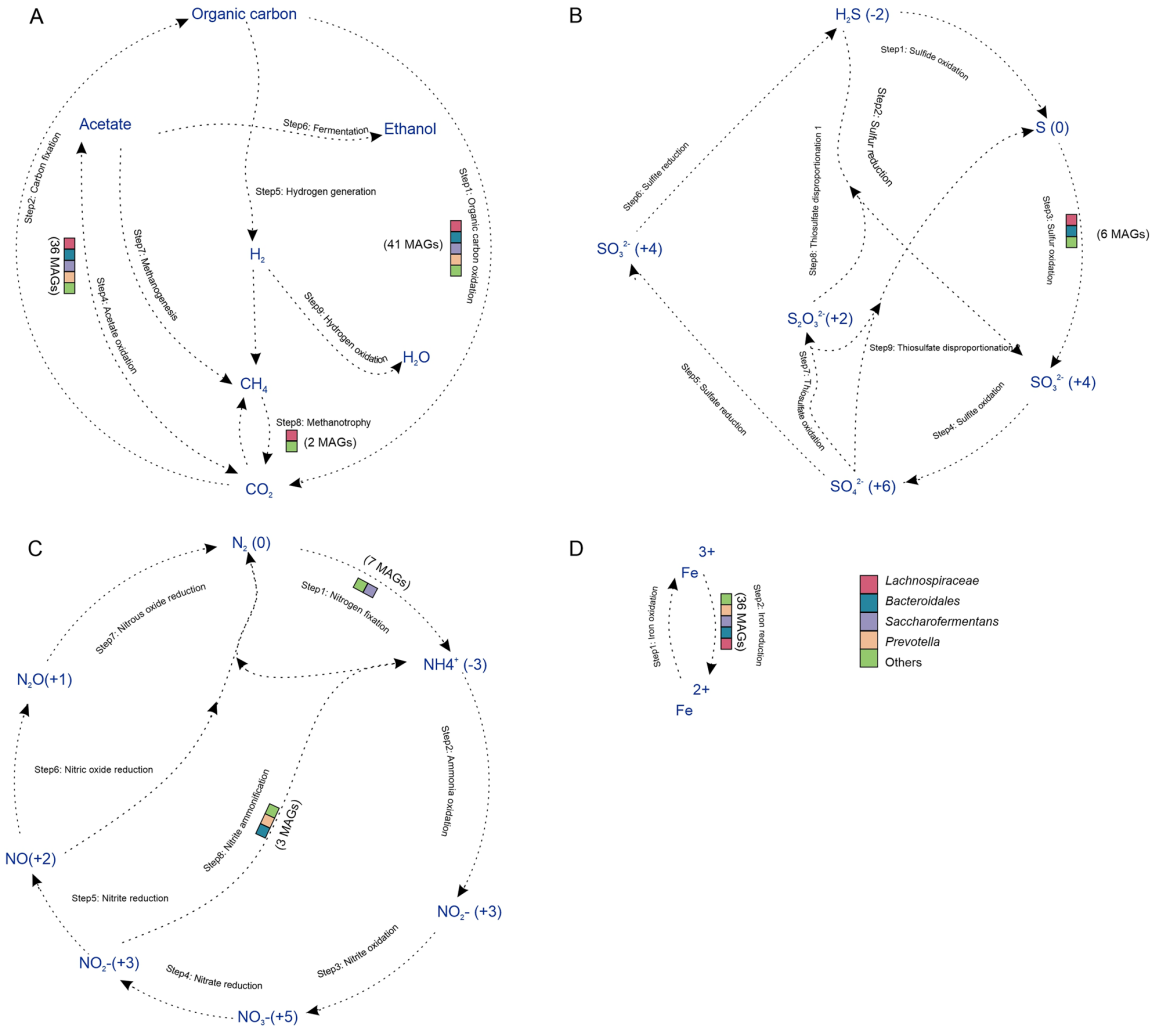
Potential biogeochemical cycling processes by rumen bacteria. Bins are color-coded in each step within the (**A**) Carbon, (**B**) sulfur, (**C**) nitrogen, and (**D**) iron cycles. Each arrow in the figure represents a single transformation step within a cycle.

Before analyzing the MGEs, we conducted a functional analysis to understand the functional characteristics encoded within metagenome-assembled genomes (MAGs) from the ruminal microbiome of Nellore cattle. For this, MAGs with completeness > 50% and those associated with MGEs were used, totaling 43 genomes (Supplementary Tables 8 and 9). This step provided essential insights into the interplay between microbial communities and the functional potential associated with MGEs in the rumen ecosystem. Our study evaluated the involvement of rumen microorganisms in these biogeochemical pathways, and we found that the majority were associated with the stages of the carbon, sulfur, nitrogen and iron cycles (Fig. 2). Regarding the carbon cycle, most of the genomes identified demonstrated involvement in the acetogenesis process and carbon degradation complex. More specifically, we identified two genomes belonging to *Lachnospiraceae* and *Bacteroidales* encoding the enzyme methane monooxygenase (mmoBD), which act in methane oxidation, and members of these clades also participated in the oxidation step of the sulfur cycle. In the nitrogen cycle, phylotypes belonging to *Saccharofermentans* and *Treponema* acted in the nitrogen fixation stage and members of the genus *Prevotella* encoded genes for the reduction of nitrite into ammonia. All taxonomic classes presented members active in the iron oxidation stage, which is fundamental for most microorganisms, due to its redox properties, being used as an electron transporter by bacteria^25,26^.

**Figure 3:**
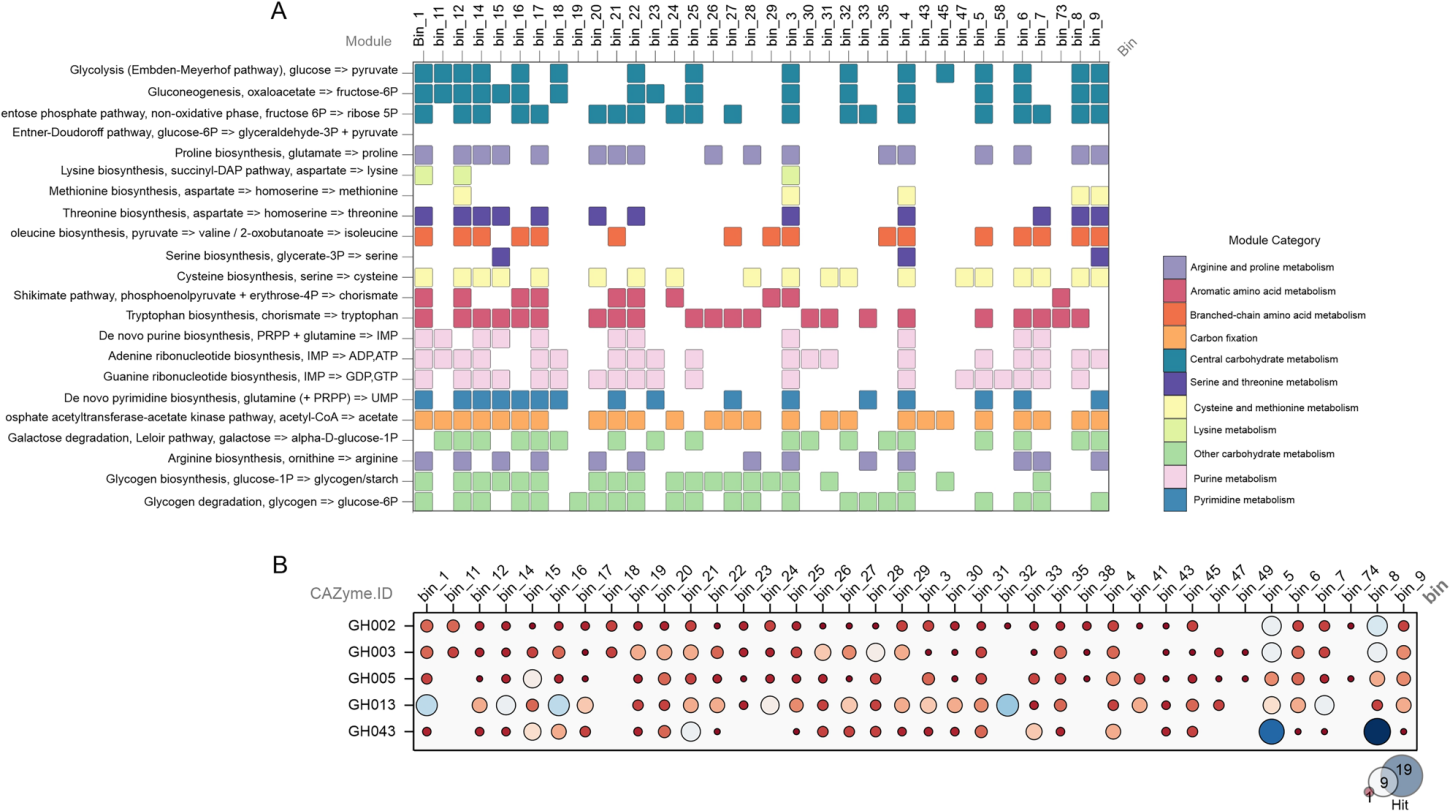
Central processes of metabolism and CAZymes present in the rumen microbiome of Nellore cattle. (**A**) Characterization of the metabolic potential of bins found in the rumen, (**B**) Identification and abundance of enzymes encoded by rumen microorganisms. The size of each circle corresponds to the number of hits for the respective gene.

The most frequent processes included steps in the central carbon metabolism pathway, the metabolism of other carbohydrates, and purine metabolism. Among the bins examined, 69% contained genes that encoded the phosphate acetyltransferase-acetate kinase pathway, a reversible process where acetyl-CoA can be converted into acetate or acetate into acetyl-CoA. Additionally, the aromatic amino acid metabolism pathway with tryptophan biosynthesis was present in 64% of the bins (Fig. 3A).

The metagenomic approach allows for the evaluation of the potential of rumen microbial populations to break down plant lignocellulolytic materials, by annotating the genes associated with this process, known as CAZymes^10^. CAZymes are classified into several categories based on their degradation capabilities, including glycoside hydrolases (GHs), carbohydrate esterases (CEs), polysaccharide lyases (PLs), and others^27^. In this study, the bins identified were assessed for their ability to encode CAZymes, and the presence of GHs and PLs was confirmed (Supplementary Table 3). We found that GH2, GH3, GH5, GH13, and GH43 were the most abundant enzymes mapped in the bins (Fig. 3B).

### Assignment of mobile genetic elements to their hosts

**Figure 4:**
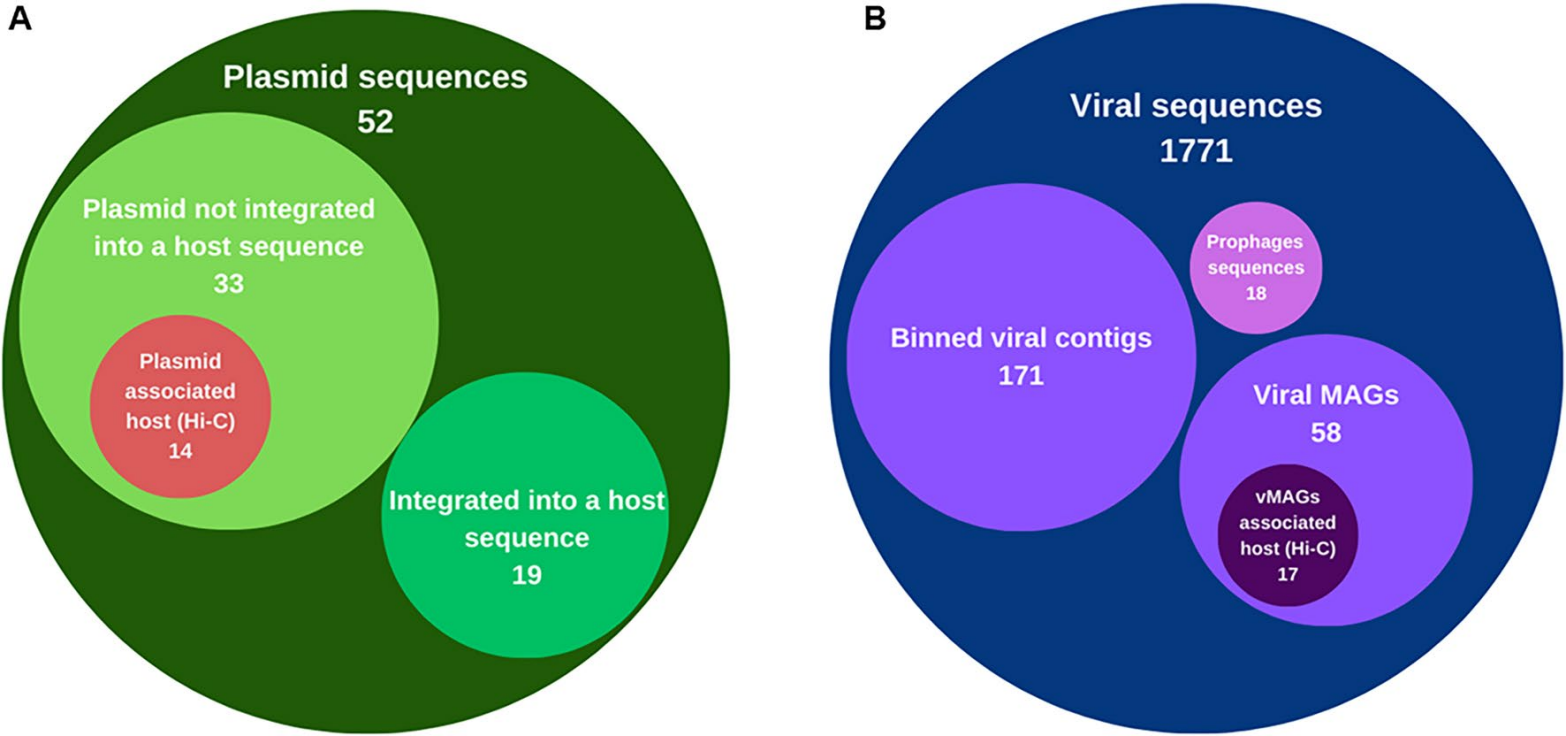
Mobile genetic elements of bovine rumen. (**A**) Each circle represents number of sequences identified as plasmids, integrated and non-host-integrated plasmids and plasmids associated with their hosts by the Hi-C method. (**B**) Each circle represents the number of identified viral sequences, viral sequences integrated into the host genome (Prophages), binned viral contigs, metagenome-assembled viral genomes (MAGs) and vMAGs associated with hosts by proximity linkage (Hi-C).

Clustering with Hi-C allows not only the construction of MAGs but also the sequence grouping of mobile genetic elements with their core genome^28^. The sequences were obtained with sequencing and the use of the proximity linkage method to assign viruses and plasmids to their microbial hosts, the data obtained is summarized in Fig. 4, more details about the data obtained are in the supplementary material (Tables 4, 6, 10, 11 and 12).

### Plasmids and the identification of microbial resistance genes

The reads derived from shotgun sequencing were used to evaluate the presence of plasmid sequences. Thirty-three DNA sequences were identified as plasmids, which were not integrated into the host genome. Among these, 14 had at least one connection with the prokaryotic host genome through Hi-C reads. Furthermore, two putative plasmids contigs k141_1760976 and k141_5289202 were shared between multiple host clusters (bin_3, bin_5, bin_20, bin_80) and (bin_1; bin_3; bin_11; bin_30; bin_88) (Supplementary Table 4). The bins shared by contig k141_1760976 belong to different genus, including *UBA2868*, *Prevotella* and *Saccharofermentans*. The plasmid k141_5289202 shared by three individuals belonging to *Lachnospiraceae* and one belonging to *Bacteroidales.*

**Figure 5:**
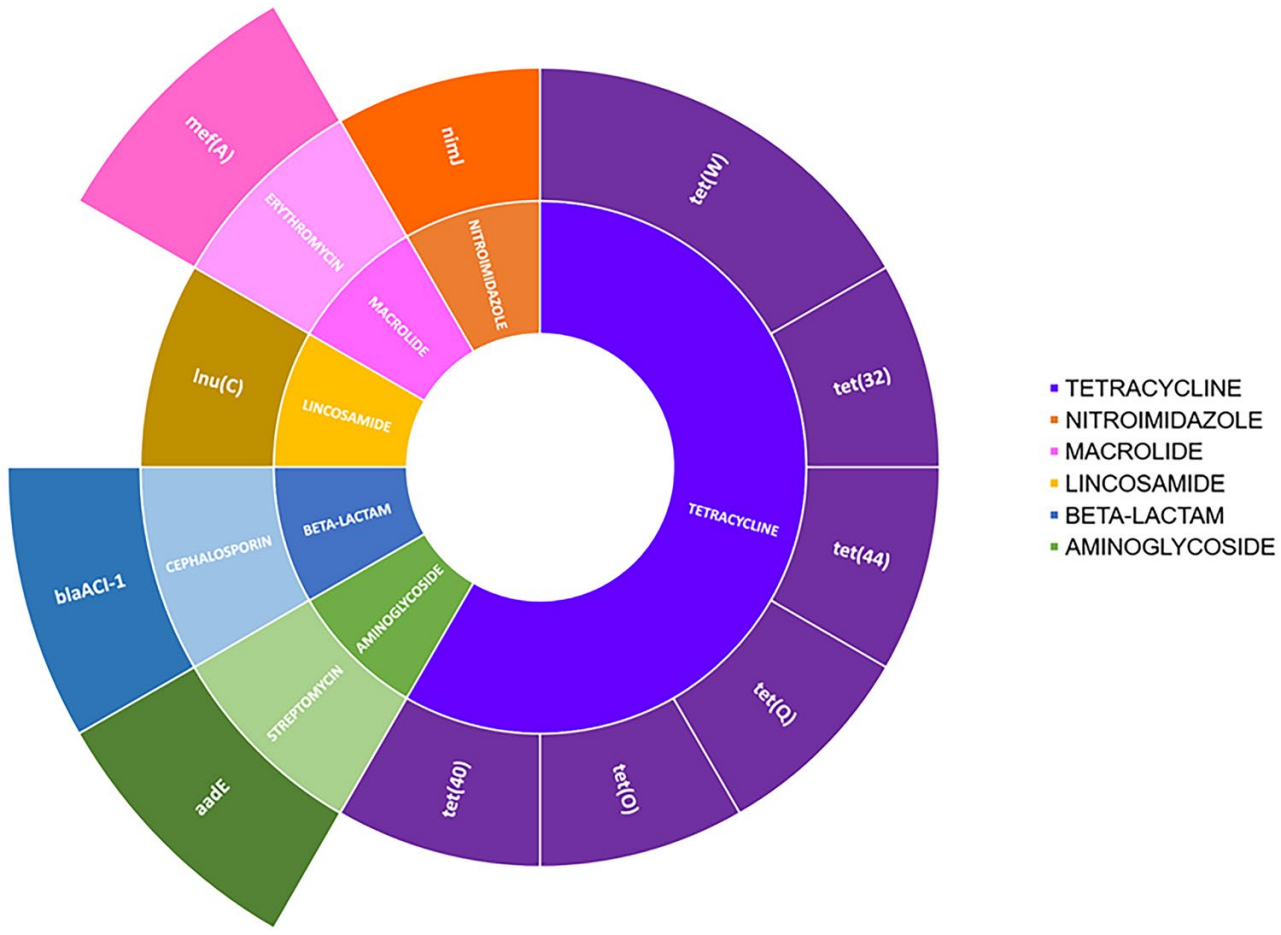
Antimicrobial resistance genes—ARGs. The information is presented hierarchically, starting from the center to the edges, beginning with the classes of antibiotics, followed by subclasses, and finally the resistance gene symbols.

Additionally, we identified 19 plasmid sequences integrated into the host genome sequences (Fig. 4A). Within this set, it was observed that 50% of the total antibiotic resistance genes (ARGs) validated in our database were present (Fig. 5). All the identified resistance genes had an identity of more than 90% with the reference sequence (Supplementary Table 5). The *tet32* gene sequence was associated with bin_14, which belongs to the *Lachnospiraceae* family.

### Virus found in the rumen

A total of 11 high-quality metagenome-assembled viral genomes (vMAGs) were identified and characterized as viral sequences (as shown in Supplementary Table 6). They had completeness rates of 92–100% and a length of 35 to 126 kbp. Another 22 MAGs were obtained with medium quality, of which 13 had completeness rates of 71–89% and a length of 25–90 kbp, and nine had completeness rates of 68–50% and lengths of 20 and 130 kbp. Any genomes with less than 50% completeness were considered low quality. All of the assembled genomes were determined to be free of contamination (Supplementary Table 6).

The majority of vMAGs had their taxonomy associated with genomes that have not yet been assigned to the ICTV phage database^29^. There were vMAGs associated with the same viral cluster (VC); however, these do not yet have taxonomic assignment in the database: vMAG_4 and vMAG_35 were associated with the viral cluster VC_146, as well as vMAG_5 and vMAGs_18, with VC_147 and vMAG_25, and vMAG_56, with VC_200 (Supplementary Table 7).

**Figure 6:**
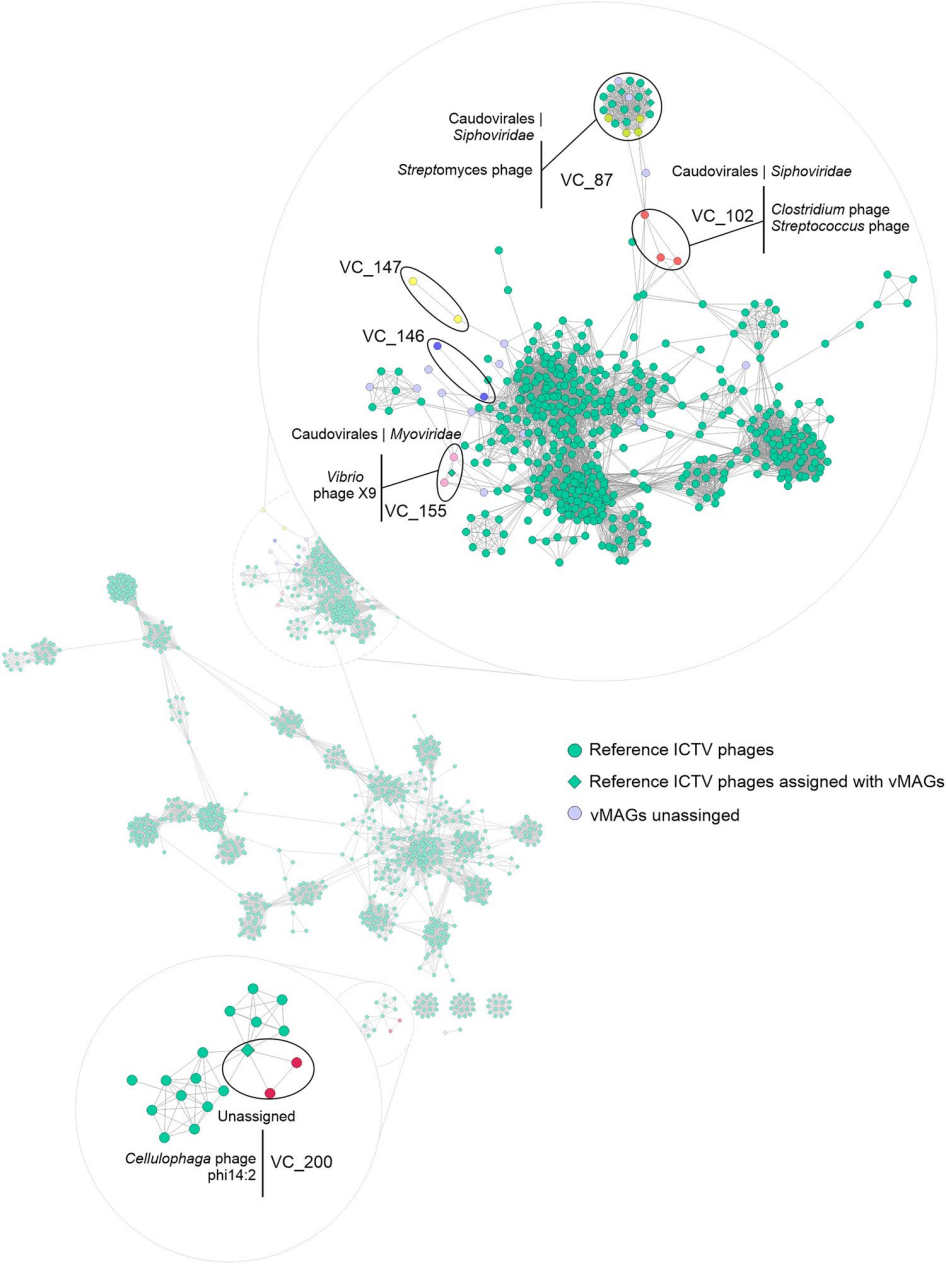
Taxonomic assignment of rumen viruses. Network of viral clusters assigned by vCONTACT2. Nodes with the same color have the same viral cluster, diamonds are clusters with known taxonomy and the nodes linked to them have the same viral cluster and can be assigned the same taxonomy, lilac nodes represent vMAGs that have not been assigned to clusters known.

Among the viruses that could be assigned to known genomes, vMAG_1 and vMAG_48 were assigned to the *Myoviridae* family VC_155, vMAG_40, vMAG_46 and vMAG_57 were assigned to the *Siphoviridae* family, VC_102, vMAG_7, vMAG_16, 29 and vMAG_52 were also assigned to the *Siphoviridae* family, but with genomes associated with *Streptomyces phage*, VC_87 (Fig. 6).

### Virus–host association

The viral clusters associated with the bacterial genomes determined by Hi-C mostly exhibited a lytic cycle. The vMAG_54, associated with the host *UBA2868 sp003535955* (*Lachnospiraceae*), presented high quality, achieved 100% completeness in its genome, and was characterized by a lytic lifestyle (Table 1).

**Table 1:** Viruses associated with bacterial hosts by Hi-C.

Name cluster host	Host	Name cluster (phage)	Life cycle	CheckV quality	Completeness (phages)
bin_30	*UBA2868 sp003535955 *(*Lachnospiraceae*)	vMAG_54	Lytic	High-quality	100.0
bin_41	*g_UBA2868* (*Lachnospiraceae*)	vMAG_13	–	Medium-quality	87.8
bin_12	*UBA2868 sp002368575* (*Lachnospiraceae*)	vMAG_4	Lytic	Medium-quality	85.14
bin_28	*Saccharofermentans sp003543635*	vMAG_45	Lytic	Medium-quality	72.67
bin_22	*UBA3766 sp902803015* (*Lachnospiraceae*)	vMAG_35	Lytic	Medium-quality	60.16
bin_30	*UBA2868 sp003535955* (*Lachnospiraceae*)	vMAG_12	Lytic	Medium-quality	54.18
bin_28	*Saccharofermentans sp003543635*	vMAG_18	Lytic	Medium-quality	50.67
bin_16	*RUG12461 sp016286115* (*Lachnospiraceae*)	vMAG_22	Lytic	Low-quality	46.05
bin_102	*Non-classified*	vMAG_43	Lytic	Low-quality	39.81
bin_27	*Butyrivibrio sp017620235* (*Lachnospiraceae*)	vMAG_17	Lytic	Low-quality	39.28
bin_58	*g_F23-D06*	vMAG_51	–	Low-quality	37.32
bin_74	*g_UBA1711* (*P3*)	vMAG_42	Lytic	Low-quality	34.54
bin_73	*Non-classified*	vMAG_27	–	Low-quality	25.64
bin_49	*Non-classified*	vMAG_6	–	Low-quality	24.02
bin_59	*RUG191 sp002373675* (*Lachnospiraceae*)	vMAG_44	Lytic	Low-quality	23.57
bin_7	*NK4A144 sp902783395* (*Lachnospiraceae*)	vMAG_24	Lytic	Low-quality	8.71
bin_26	*Saccharofermentans sp902782235*	vMAG_49	Lytic	Not-determined	NA

g_(genus).

**Figure 7:**
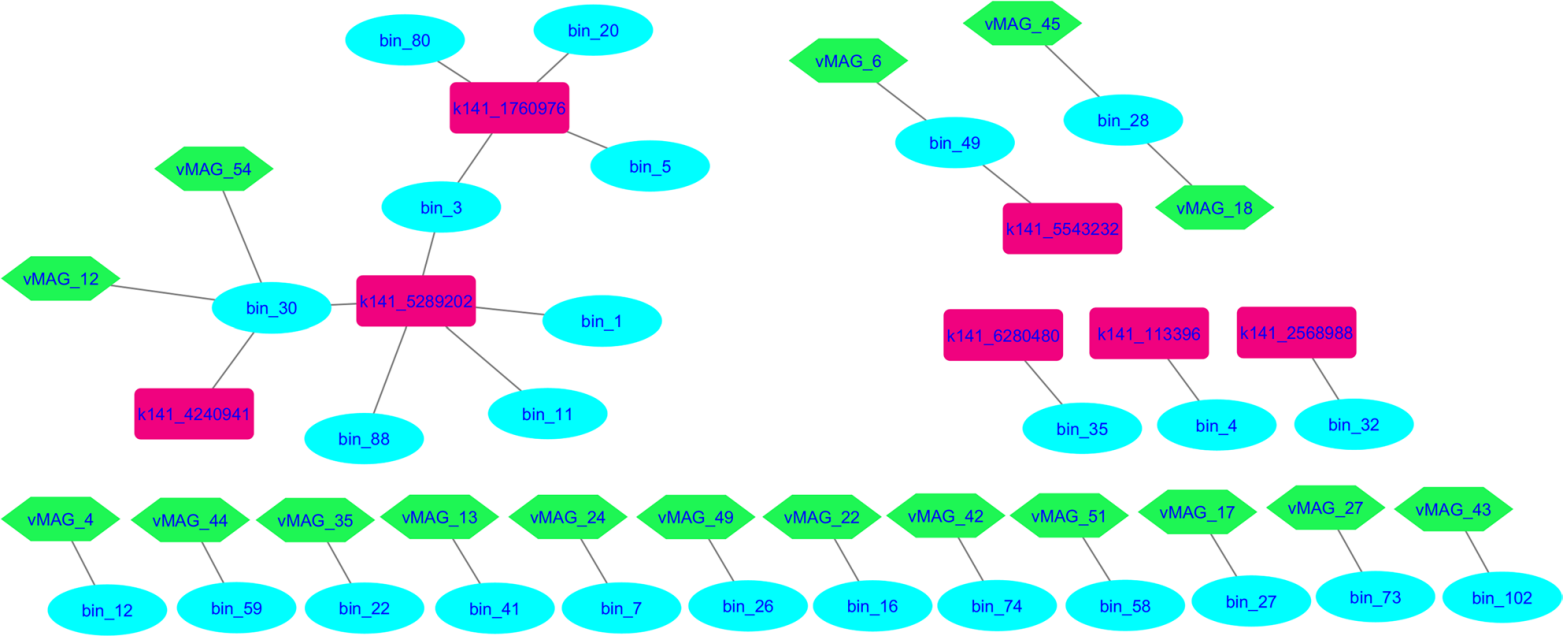
Association between bacterial hosts and their Hi-C-linked mobile genetic elements. Ellipses represent bacterial hosts, hexagons represent bacteriophages, and rectangles represent plasmids.

Two co-infection events were identified, in which bacterial cells presented the assignment of more than one bacteriophage simultaneously, that is, different bacteriophages infecting the same bacterial host. Bacteriophages corresponding to vMAGs 18 and 45 infected the same bacterial cell corresponding to bin_28, and the same occurred with vMAGs 12 and 54, which infected the same bacterial cell corresponding to bin_30 (Fig. 7).

Of the 17 bins identified to be associated with viruses (Supplementary Table 12), 8 belonged to the *Lachnospiraceae* family. The bin_30 and bin_41 belong to the same genus *UBA2868*, and the other bins assigned to this family were: (bin_7) *NK4A144 sp902783395*, (bin_12) *UBA2868 sp002368575*, (bin_16) *RUG12461 sp016286115*, (bin_22) *UBA3766 sp902803015*, (bin_27) *Butyrivibrio sp017620235,* and (bin_59) *RUG191 sp002373675*. The bin_26 and bin_28 were assigned to *Saccharofermentans sp902782235* and *sp003543635*, respectively, and bin_58 was assigned to genus *F23-D06* and bin_74 to genus *UBA1711*, both belonging to the order *Bacteriodales*.

## Discussion

Metagenomics has enabled the efficient identification of microorganisms, enzymes, and metabolic pathways that play a role in plant breakdown in the bovine rumen^30^. This tool has also proven to be crucial for identifying the microbiota and characterizing important traits such as methane production and feed efficiency^3,8,31^. This study is the first to explore rumen mobile genetic elements of Nellore cattle fed a grass diet by associating viruses and plasmids with their hosts through physical links between DNA molecules from the same genome using the Hi-C method^22^. We identified 31 links between bacteria and mobile genetic elements, co-infection events and genes conferring antibiotic resistance. Additionally, we assembled a collection of 34 bacterial genomes with completeness > 50%, which mostly encoded pathways for central carbon and other carbohydrate metabolisms.

In this study, we identified 52 plasmid sequences, 14 of which were linked to their hosts using Hi-C. Similarly, in their evaluation of the canine fecal metagenome, Cuscó et al.^24^ found that six Hi-C plasmids were ligated into their bacterial hosts, including five circular plasmids and a plasmid carrying the linA resistance gene. In our investigation, we uncovered two plasmids, k141_1760976 and k141_5289202, which are associated with multiple host clusters. As previously reported by Mo et al.^32^ and Stewart et al.^28^, sequences may be shared among various bacterial species found in the rumen. Through the Hi-C approach, Stalder et al.^33^ found that plasmids are efficient vectors for horizontal gene transfer and that this method is useful for tracing microorganisms that harbor antibiotic-resistant genes. We identified 12 genes conferring antibiotic resistance, and among these, six genes were found to confer resistance to tetracycline: *tet32*, *tet40*, *tet44*, *tetO*, *tetQ*, and *tetW*. These findings are consistent with a previous study by Jing and Yan^34^, in which tetracycline resistance genes (*tet44*, *tetQ*, and *tetW*) were the most prevalent in rumen content samples and were the most abundant in the genome of *Prevotellaceae*, but *Lachnospiraceae* were also present. Furthermore, *tetQ*, *tetW*, and *tet40* were also detected in most samples derived from the rumen of 48 beef cattle from three different taurine breeds, the expression of these genes was associated with the stability of the microbiota, which may give it greater resistance to external perturbations such as diet, for example^35^.

In addition to the genes that confer resistance to tetracycline, other classes of antibiotics, such as nitroimidazole *nimJ*, macrolide *mefA*, lincosamide *lnuC*, beta-lactam *blaACI-1*, and aminoglycoside *aadE*, were also identified. Genes that confer resistance to macrolides, lincosamide, and aminoglycosides were the most prevalent among ARGs in the study conducted by Ma et al.^35^, with the *mefA* gene being one of the most common. Additionally, ARGs associated with plasmids were identified, indicating that plasmids should be the focus of future investigations on antimicrobial resistance in livestock. Auffret et al.^36^ also found an increase in ARGs in rumen content samples from cattle fed diets with higher concentrate content, with an abundance of genes related to resistance to macrolides and beta-lactams, suggesting that this increase is associated with dysbiosis caused by more concentrated diets. The findings of this study are in agreement with those of an earlier in silico study conducted by Sabino et al.^37^, who analyzed the rumen resistome in 435 microbial genomes and found a high prevalence of antibiotic resistance genes (ARGs) that provide resistance to β-lactams, glycopeptides, tetracyclines, and aminoglycosides. Recent metagenomic studies have indicated that bovine rumen is a significant contributor to antibiotic resistance^34^, it is not necessarily related to the administration of antibiotics, considering that the rumen contents of young animals that did not receive antibiotic treatment demonstrated the presence of ARGs^38,39^.

Advancements in bioinformatics have contributed to a deeper understanding and better characterization of the rumen virome. Yan et al.^11^ identified 397,180 viral operational taxonomic units (vOTUs) from 975 metagenomes of 13 ruminant species, with the majority of metagenomes derived from taurine animals. Only 23 metagenomes were from *Bos indicus* raised in Kenya, and none were from Brazil, supporting the significance of this research. The viral metagenomic assemblages discovered in this study were mostly assigned to unclassified genomes, which is probably because of the limited rumen virome of *Bos indicus* data in the ICTV phage database. This finding is consistent with those of Sato et al.^40^, who observed low numbers of viral operational taxonomic units shared with the RefSeq database for samples from the rumen of Japanese cattle. The assigned vMAGs belong to the families *Myoviridae* and *Siphoviridae*, which are commonly found in the rumen environment and constitute the most abundant group, along with *Mimiviridae* and *Podoviridae*^13,16,40,41^.

A group of bacteria classified as *UBA2868 sp003535955* (unclassified *Lachnospiraceae bacterium*, NCBI) was found to have the highest percentage of complete vMAGs (100%) and was characterized as high and medium quality. This genus was previously identified in a community of uncultured microorganisms from the intestines of pigs through sequencing of fecal samples^42,43^. Bacteria from the unclassified family *Lachnospiraceae* are prevalent in the ruminal environment and serve as the central microbiome in cattle^9^. This allowed us to infer that population-associated viruses play a crucial role in the bovine rumen, rather than populations present in low abundance, which remains a challenge^21^.

Friedersdorff et al.^44^ successfully isolated and sequenced active lytic phages belonging to the *Siphoviridae* family that infect *Butyrivibrio fibrisolvens*, which was found in both the rumen and feces of cattle and sheep. We discovered a lytic phage in *Butyrivibrio sp017620235*, which is commonly found in the rumen and promotes the degradation of lignocellulose and fermented carbohydrates into butyrate, formate, lactate, and acetate^45–47^. Two bacterial species classified as Rumen Uncultured Genomes (RUGs), (bin_16) *RUG12461 sp016286115* and (bin_59) *RUG191 sp002373675*, were also associated with viruses having a lytic life cycle.

The study's assembly bacterial genomes achieved a high completeness (> 95%) for members of the *Treponema* genus, which are known as spirochetes and have been positively correlated with feed conversion in cattle^3^. These species are considered to be one of the main fiber degraders in the rumen of Gir cattle, along with other bacteria, such as *Clostridium*, *Ruminococcus*, *Eubacterium*, *Butyrivibrio*, *Roseburia*, *Caldicellulosiruptor*, and *Rhodospirillum*^48^. The bins identified were primarily assigned to families, such as *Lachnospiraceae*, *Bacteroidaceae*, *P3*, *Ruminococcaceae*, *Saccharofermentanaceae*, and *Treponemataceae*. These bins mainly encoded pathways related to central carbon metabolism and metabolism of various carbohydrates. One notable pathway found between these bins is the phosphate acetyltransferase-acetate kinase pathway, which is activated at high acetate concentrations, requires less ATP, and plays a role in energy metabolism^49^. Additionally, the aromatic amino acid metabolism pathway with tryptophan biosynthesis was abundant among the bins in the current study. This pathway occurs only in microorganisms and plants, and tryptophan is obtained by animals via symbiosis for protein synthesis^50^.

The identification of CAZymes in the bacterial genomes revealed the presence of genes encoding glycoside hydrolases and polysaccharide lyase groups, with GH2, GH3, GH5, GH13, and GH43 being the most prevalent. These groups degrade xylan polysaccharides, which are heterogeneous and require different catalytic enzymes^10^. In a study by Wang et al.^51^, the same GH families, GH2, GH3, GH13, and GH43, were found to comprise the majority of CAZymes present in the rumen of dairy cows fed different proportions of roughage and concentrate. The GH3 group was particularly more abundant in the high-roughage diet, and these observations are consistent with the current study's findings for pasture-fed Nellore cows, where glycoside hydrolase groups were the most prevalent.

In summary, this study provides a thorough examination of the MGEs in the rumen of Nellore cattle, shedding new light on the connection between these elements and their microbial inhabitants. The impact and function of these MGEs in beef cattle are currently being investigated by our research group, we recognize the need to validate the results of the present study in a significant number of animals to better understand the ruminal dynamics of Nellore cattle kept at pasture.

## Material and methods

### Animal characterization and sample collection

Samples of ruminal content were collected from four Nellore (*Bos taurus indicus*) nulliparous cows cannulated in the rumen, with an average weight of (600 ± 50 kg) and age between 6 and 9 years, from the Ruminant Nutrition Laboratory, Faculty of Veterinary Medicine and Animal Science, FMVZ, located on Pirassununga (SP), in Brazil. The experimental protocols were approved by Ethics Committee on the Use of Animals of Faculty of Veterinary Medicine and Animal Science (CEUA/FMVZ) under number 7333211118. The experimental protocols were conducted in accordance with the relevant guidelines and regulations. The study design and analysis conform to the ARRIVE recommendations for animal research. The animals received a diet exclusively based on *Brachiaria brizantha* pasture and mineral salt ad libitum, which was used only for animal maintenance (Table 2) for approximately four months before the ruminal content collection. Samples were kept on ice during collection until processing and were subsequently stored at − 80 °C.

**Table 2:** Chemical composition of the diet.

Diet	Content
*Brachiaria brizantha*	Ad libitum
Chemical composition (% DM)	
Dry matter	34.1
Ether extract	1.9
Crude protein	6.9
Neutral detergent fiber	70.5
Mineral salt^1^	Ad libitum

^1^Composition of mineral salt per kilogram of product: 160 g of Ca, 80 g of P, 30 g of S, 155 g of Na, 100 mg of Co, 1.250 mg of Cu, 62.5 mg of I, 1.125 mg of Mn, 25 mg of Se, 3.750 mg of Z.

### DNA extraction and sample preparation for Hi-C

DNA extraction was performed using a QIAmp Fast DNA Stool Mini Kit (Qiagen, USA). The crosslinking procedure was performed, in which the samples were processed according to the protocol recommended by the company Phase Genomics (Seattle, WA, USA) adapted by Burton et al.^52^. Briefly, the sample containing liquid and fiber (5 ml) were suspended in 1% formaldehyde (45 ml) and incubated (20 min) at room temperature with periodic shaking. Subsequently, glycine (1 g/100 ml) was added, which was then incubated (15 min) at room temperature with periodic shaking. The sample was centrifuged (1000×*g* for 1 min) and washed with PBS. It was then centrifuged again and the supernatant was removed. The pellets obtained formed a pool and were transferred to a cryotube (2 ml), stored at − 80 °C until sent along with the DNA extracted from the same sample, to be processed and sequenced by Phase Genomics.

### Hi-C library preparation and short-read sequencing

To prepare Hi-C libraries, the Phase Genomics ProxiMeta Hi-C kit v4.0 was used as described by the manufacturer^53^. After going through the crosslinking process, the pellets underwent lysis and their DNA–protein complexes were digested by the restriction enzymes (Sau3AI and MlucI)^32^, creating free ends on the DNA strands that had labeled nucleotides with biotin to create chimeric molecules composed of fragments from different physically close genome regions in vivo. Proximity-linked DNA molecules were drawn down using streptavidin beads and processed into an Illumina-compatible sequencing library.

Separately, DNA was extracted from an aliquot of the original sample, and a shotgun metagenomic library was prepared using ProxiMeta library preparation reagents. Sequencing was performed using Illumina NovaSeq, generating pairs of PE150 reads for the Hi-C and shotgun libraries. Hi-C and shotgun metagenomic sequencing files were uploaded to the Phase Genomics cloud-based bioinformatics portal for subsequent analyses.

### Clustering, quality assessment, taxonomic and functional assignment of assembled microorganisms

Shotgun-derived reads were filtered and adjusted for quality, normalized using fastp^54^, and assembled with MEGAHIT^55^ using default options. Pairs of Hi-C reads were aligned to each assembly using BWA-MEM^56^ with the -5SP options specified and all other default options to disable attempts to pair the reads according to the normal Illumina settings. The SAMBLASTER program^57^ was used to flag PCR duplicates that were subsequently excluded from the analysis. The alignments were then filtered with SAMtools^58^ using the -F 2304 filter flag to remove nonprimary and secondary alignments.

Deconvolution, a method that uses the intracellular proximity signal captured by Hi-C as an indicator of the cellular origin of metagenome sequences, was performed using ProxiMeta^28,32^ to generate putative genomes and clusters of genome fragments. Prokaryotic clusters were assessed for quality using CheckM^59^ and received preliminary taxonomic classifications using Mash, a generalized and automated method that identifies reference genomes compatible with the NCBI RefSeq database^60^ (Supplementary Table 1). Subsequently, the bins were submitted to the Genome Taxonomy Database (GTDB)^61^ for taxonomic assignment (Supplementary Table 2).

The functional potential of the bins was evaluated by METABOLIC (METabolic and BiogeOchemistry analyses in microCrobes)^62^, which detected the presence or absence of KEGG modules for the most abundant central pathways present in the genome of the microorganisms evaluated, as well as how they attributed the CAZymes and the main biogeochemical cycles present.

### Mobile genetic elements

Viral contigs were annotated and used for lifestyle and viral protein annotation per contig using VIBRANT^63^. The contigs were grouped into assembled genomes using ProxiPhage viral binning. Sequence integrity was validated with CheckV, viral genomes were classified into quality levels based on AAI or HMM estimation, high confidence (≥ 90% complete), medium confidence (80–90% complete) or low confidence (< 80% complete)^23,64^. For taxonomic assignment, the protein sequences of the viral clusters (vMAGs) were predicted using Prodigal^65^, and these sequences were used to build protein cluster profiles that were compared with sequences present in the ICTV phage database, generating networks of similarities between viral clusters with the using vCONTACT2^66^.

To identify sequences with plasmid characteristics, a BLAST (Basic Local Alignment Search Tool) alignment was performed against the NCBI plasmid database^32^. Sequences annotated as plasmids were associated with the host using a host-finding algorithm^23^. Microbial resistance genes, in turn, were identified with AMRFinderPlus, which allows the assessment of Antimicrobial Resistance gene content in the NCBI database^67^.

### Assignment of mobile genetic elements to their hosts

The step of assigning mobile genetic elements to their hosts was composed of several filtering criteria, with the first phase of filtering restricting connections with less than 2 Hi-C read links. To remove false positives, a connectivity rate (≥ 0.1) was used for the number of copies of mobile elements per cell (for repeated vMAGs the highest number of counts per cell was considered) and host intra-MAG connectivity (≥ 10 links). Finally, a receiver operating characteristic (ROC) curve was performed, which determines the ideal copy count cutoff value (≥ 0.1). This value is expected to be close to 1, as values close to or above 1 are considered reliable for attributing a real host^23^. In cases where the optimal copy count threshold is excessively low (< 0.1), it is replaced with 0.1. This occurred in the present study, where the calculated value was 0.01.

### Supplementary Information

Supplementary Tables.

## Data Availability

The data generated from the present study will be available in the Sequence Read Archive (SRA) database https://dataview.ncbi.nlm.nih.gov/object/PRJNA1054691?reviewer=vd7njv2i8dqvoj4d6tmt82kr1t.
